# Transcatheter aortic valve implantation: from fantasy to reality

**DOI:** 10.1186/1749-8090-9-43

**Published:** 2014-03-07

**Authors:** Kasra Shaikhrezai, Billy McWilliams, Edward T Brackenbury, Sai Prasad, Tristan D Yan, Renzo Pessotto, Vipin Zamvar, Geoffrey Berg

**Affiliations:** 1 Department of Cardiothoracic Surgery, Golden Jubilee National Hospital, Glasgow, Clydebank G81 4DY, UK; 2 Department of Anaesthetics, Intensive Care & Pain Management, Cardiff University, Cardiff, UK; 3 Department of Cardiothoracic Surgery, Royal infirmary of Edinburgh, Edinburgh, UK; 4 Department of Cardiothoracic Surgery, Royal Prince Alfred Hospital, Sydney, Australia

**Keywords:** Transcatheter Aortic Valve Implantation (TAVI), Technology, Evidence-based practice

## Abstract

Increased life expectancy has led to the presentation of more complicated patients in old age for the replacement of the aortic valve. The emergence of Transcatheter Aortic Valve Implantation (TAVI) was considered as a significant breakthrough in the management of symptomatic, moribund patients suffering from aortic valve stenosis who had been rejected for surgical intervention. A novel technology often has a long journey from the point at which it is created to its every-day-use. It is now obvious that TAVI practice in multiple institutes around the world has gone beyond the evidence. Serious concerns have been raised questioning the current TAVI practice. Analysis of future TAVI use may assist clinicians and healthcare managers to understand and deploy this technology in accordance with the evidence.

## Background

In the spring of 2002 Alain Cribier deployed the first transcatheter valve implantation in a moribund patient who had been rejected for surgery [1]. In January 2004 Edward Lifesciences (Inc. Irvine, CA, USA) began mass production of catheters over which an expandable valve can be driven into the aorta up to its anatomical position at the aortic root [2].

Traditionally a new technology in medicine is evaluated on the basis of its safety, efficacy and effectiveness. In addition Markov model can be used for economical evaluations [3]. We did not examine the TAVI technology according to the aforementioned criteria and tools while our main focus was to analyse the TAVI trends by using the Gartner Hyper curve [4].

The literature was searched via Medline using the OVID interface and where appropriate the level of evidence is mentioned according to the Oxford Centre for Evidence-based Medicine (OCEBM) [5] classification.

### TAVI gartner hype curve

**Figure 1:**
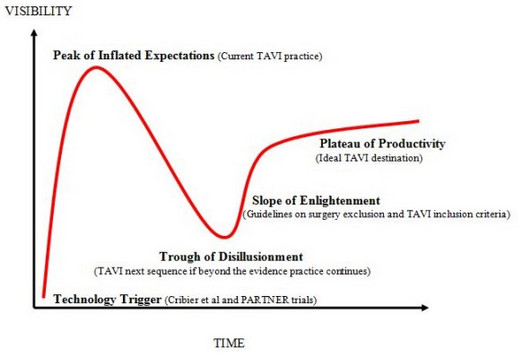
TAVI Gartner-Hype curve.

In 1995 the Gartner Company (Inc., Stamford, CT, USA), an information technology research and development corporation introduced a new tool called “Hype” cycle to analyse the behaviour of any emerging technology and assist organisations and investors to predict the technology trends [3]. As TAVI has been certified and recognised as a technology [6] (level 5), it is appropriate to analyse its behaviour since creation using the Hype cycle phases (Figure 1).

#### Phase 1: technology trigger

TAVI did not reach the stage of product launch until Edward Lifesciences (Inc., Irvine, CA, USA) produced SAPIEN® valves; closely followed by the Medtronic CoreValve® (Medtronic Inc, MN, USA). The product launch was disseminated by Leon et al. [7] (level 1B), who first published the results of a randomised controlled trial comparing TAVI vs. medical therapy/valvuloplasty vs. Aortic Valve Replacement (AVR). The results of PARTNER studies [7-10] (level 1B) have been widely used for of the promotion of TAVI programmes and significantly influenced the management of high-risk patients [11] (level 5). These studies demonstrated that in selected high risk patients suffering from aortic stenosis the survival in 1-year was similar in TAVI and AVR. It was also concluded that at 2-year follow-up TAVI is an alternative to AVR in high risk patients who were not suitable candidates for surgery.

The guideline published recently by the joint task force on the management of valvular heart disease of the European Society of Cardiology (ESC) and the European Association for Cardio-Thoracic Surgery (EACTS), has also recommended TAVI for high risk patients who are not suitable for surgery [12].

#### Phase 2: peak of inflated expectations

This phase was reached following TAVI interventions conducted throughout the world; this recently exceeded 40,000 procedures [13]. In fact, TAVI may reach the peak of the Hype cycle in a relatively short period of time owing to the enthusiasm of pro-TAVI clinicians and industry marketing pressures. Germany, as the largest consumer of this technology in Europe [13], did not have a reliable registry until the creation of the German Aortic Valve Registry (GARY) in 2010 [14], implying a haphazard recruitment of patients for TAVI on a large scale. In Canada, 6 years prior to United States Food and Drug Administration (FDA) approval, the TAVI programme was limited to patients on compassionate grounds and recruited a number of patients within four years. Although it has been demonstrated that TAVI is in consistence with post-operative paravalvular regurgitation and stroke, the TAVI programme continued to recruit larger number of patients by expanding the criteria and lowering the bar of risk stratification. Concerns have been expressed that potential TAVI candidates constitute a widely heterogeneous group of patients [15] (level 4); this may directly influence a bias-free patient recruitment for TAVI programmes. Retrospective analysis of outcomes of conventional AVR in high-risk patients who are also potential candidates of TAVI, demonstrated that widening of the inclusion criteria for TAVI may be inappropriate [16] (level 4).

Currently, there are no clear guidelines available to assist the surgeons in determining which patients would best be treated by TAVI or AVR. In 2012 a group of independent researchers [6] claimed that more than 40,000 TAVI procedures which have been performed throughout the world cannot be justified from both a clinical and cost-effectiveness point of view. The analysis criticises the clinicians who have manipulated the indications to beyond the evidence in TAVI practice.

Neil Moat, the principle investigator of the first major UK TAVI registry [17], says “We have enough experience with TAVI now that we have to accept that the devices are different and they do have different advantages and disadvantages, and I think it’s excellent that we are starting to discuss the type of patients that would benefit from one device or another” [13]. This statement also confirms that the TAVI technology is currently at its peak of inflated expectations moving towards the “Trough of disillusionment” phase*.*

#### Phase 3: trough of disillusionment

This phase has not yet arrived for TAVI. In this sequence the use of the technology visibly diminishes indicating that it has just become unfashionable. In this stage the rate of publication of TAVI-related articles topics reduces significantly.

#### Phase 4: slope of enlightenment

The Scottish Government report on TAVI [18] (level 5) confirms that: “There is no consensus on what constitutes high surgical risk, no reliable method to identify elderly patients are most likely to benefit from AVR and no standard criteria by which to select patients for TAVI.” Although the report confirmed that TAVI must be only used in patients who are considered as inoperable it concluded: “There is a lack of standardisation of the definitions of “inoperable” and “high risk” as they are primarily based on clinical judgement.” The report also revealed that the minimum cost of the procedure is £21,059 whereas for AVR is much less. The report concluded that TAVI for inoperable patients is more expensive yet, at the same time, more effective than medical therapy. However there is a paucity of evidence with regards to economic burden of TAVI.

In 2011 Hartzell Schaff in the editorial section of the New England Journal of Medicine [19] (level 5) raised his concerns regarding the large risk of stroke in TAVI patients which can be up to 8.3% in 1 year [8]. He also noted that TAVI does not remove the disease therefore the diseased valve may create an irregular zone which makes the patient vulnerable to thromboembolic events. Alain Cribier has also expressed concerns regarding the extensive deployment of this technology without a sufficient follow-up on the durability. He says: “I have to continually fight against a tendency to treat patients who are good surgical candidates with TAVI. The issue is that, the long-term durability of the TAVI valve is unknown, but the surgical technique lasts for 20 years” [2].

In this phase the true understating of TAVI technology needs to be achieved. This includes the TAVI capability with transparent advantages and disadvantages.

#### Phase 5: plateau of productivity

In the guideline published by the National Institute for Health and Clinical Excellence [20] it was emphasised that there is sufficient evidence of serious complications of TAVI. Although the NICE guideline clearly states that TAVI is the treatment of choice for patients not suitable for surgery, it does not clarify the definite contraindications to conventional surgery. It is sometimes the case that a surgeon’s decision to deny a patient conventional surgery, has been altered by another surgeon who subsequently operated upon the same patient with satisfactory outcomes. It has been previously demonstrated that AVR rejection constitutes a high degree of subjectivity [21] (level 4).

At this stage a consensus on the actual use of TAVI in daily practice needs to be achieved in order to minimise the bias and subjectivity of decision making process. Both latter phases would be achieved when a standardised protocol is available by which the high risk patients can be precisely rejected for surgery.

## Conclusion

When Percutaneous Coronary Intervention (PCI) took over the Coronary Artery Bypass Graft (CABG) practice in a large number of patients it was not predictable that coronary stenting will soon go beyond the evidence and even reach to malpractice [22]. The warning given by Van Brabandt et al. [6] is important to avoid the mistakes of PCI practice and adhere to the evidence in promoting the TAVI programme. Hype cycle can be used as a road map to facilitate the progress of TAVI as an adjunct to AVR to treat moribund inoperable patients and aid healthcare planning.

Multidisciplinary approach is the foundation of TAVI practice and the “heart team” comprising the cardiologists, surgeons and anaesthetists must be the core of the practice for all referred patients.

## Abbreviations

AVR: Aortic valve replacement; CABG: Coronary Artery Bypass Graft; ESC: European Society of Cardiology; EACTS: European Association for Cardio-Thoracic Surgery; FDA: United States Food and Drug Administration; GARY: German Aortic Valve Registry; NICE: National Institute for Health and Clinical Excellence; OCEBM: Oxford Centre for Evidence-based Medicine; PARTNER: Placement of aortic transcatheter valves; PCI: Percutaneous coronary implantation; TAVI: Transcatheter aortic valve replacement.

## Competing interests

The authors declare that they have no competing interests.

## Authors’ contributions

KS conducted the literature search and wrote the manuscript; BM supervised the project and revised the manuscript; ETB revised the manuscript and recommended papers; SP revised the manuscript and recommended papers; TY revised the manuscript and recommended papers; RP revised the manuscript and recommended papers; VZ revised the manuscript and recommended papers; GB supervised the project, revised the manuscript and recommended papers. All authors read and approved the final manuscript.
